# Long-Term Dynamics of SARS-CoV-2 Variant-Specific Neutralizing Antibodies Following mRNA Vaccination and Infection

**DOI:** 10.3390/v17050675

**Published:** 2025-05-06

**Authors:** Veronika Vaňová, Jana Náhliková, Martina Ličková, Monika Sláviková, Ivana Kajanová, Ľubomíra Lukáčiková, Miroslav Sabo, Žofia Rádiková, Silvia Pastoreková, Boris Klempa

**Affiliations:** 1 Institute of Virology, Biomedical Research Center, Slovak Academy of Sciences, 845 05 Bratislava, Slovakia; veronika.vanova@savba.sk (V.V.); jana.nahlikova@savba.sk (J.N.); martina.lickova@savba.sk (M.L.); monika.slavikova@savba.sk (M.S.); viruivvi@savba.sk (I.K.); lubomira.lukacikova@savba.sk (Ľ.L.); silvia.pastorekova@savba.sk (S.P.); 2 Institute of Experimental Endocrinology, Biomedical Research Center, Slovak Academy of Sciences, 845 05 Bratislava, Slovakia; miroslav.sabo@savba.sk; 3 Institute of Clinical and Translational Research, Biomedical Research Center, Slovak Academy of Sciences, 845 05 Bratislava, Slovakia; zofia.radikova@savba.sk

**Keywords:** SARS-CoV-2, Pseudotypes, neutralization assays, antibodies, longitudinal analysis

## Abstract

Understanding the long-term dynamics of SARS-CoV-2 neutralizing antibodies is critical for evaluating vaccine-induced protection and informing booster strategies. In this longitudinal study, we analyzed 114 serum samples from 19 individuals across six time points over a three-year period following mRNA vaccination (Comirnaty) and natural SARS-CoV-2 infection. Using pseudotype-based neutralization assays against nine SARS-CoV-2 variants, including major Omicron subvariants (BA.1–BA.5, BQ.1.1, XBB), and anti-S1 IgG ELISA, we observed that antibody levels peaked after the third vaccine dose and remained relatively stable two years later. Neutralization titers rose markedly after the second and third doses, with the highest neutralization observed at two years post-booster. Strong correlations were found between anti-S1 IgG levels and mean neutralization titers for pre-Omicron variants (r = 0.79–0.93; *p* < 0.05), but only moderate for Omicron subvariants (r ≈ 0.50–0.64). Notably, hybrid immunity (vaccination plus infection) resulted in higher neutralization titers at the final time point compared to vaccine-only participants. The lowest neutralization was observed against XBB, underscoring the immune evasiveness of emerging variants. These findings support the importance of booster vaccination and highlight the added durability of hybrid immunity in long-term protection.

## 1. Introduction

The emergence of the novel SARS-CoV-2 virus in late 2019 introduced an unprecedented challenge to global public health, as the human immune system had never encountered this pathogen before [1]. This led to a rapid and intense worldwide response, with scientists and healthcare institutions collaborating to develop effective vaccines. These vaccines have played a crucial role in curbing the pandemic, significantly reducing infection rates and mortality from COVID-19 [2]. Nonetheless, the virus’s rapid mutation capability leads to the development of new variants, as evidenced by the Delta wave and subsequent Omicron waves, allowing it to evade the immune response generated by vaccination or prior infection [3]. Thus, reinfection with the virus is possible. However, immunization and previous infection with SARS-CoV-2 have established immunological memory, including neutralizing antibodies (NAbs), which can be reactivated upon reinfection, resulting in less severe disease [4].

Immune memory consists of several components that act in concert to prevent reinfection and severe disease. Memory CD8 T cells eliminate virus-infected cells, while CD4 T cells support both CD8 T cell and B cell responses. Memory B cells produce high-affinity antibodies upon re-exposure to the virus, and circulating antibodies—mainly IgG and IgA—neutralize the virus before it enters cells [4,5,6,7]. These antibody responses involve distinct isotypes (IgG, IgA, and IgM), each with different kinetics and durability [8,9,10,11]. Their longevity is influenced by antigen type (spike vs. nucleocapsid), infection severity, and age [7,12,13].

The SARS-CoV-2 virus is a positive-sense RNA virus that encodes 29 proteins, both structural and nonstructural, including the spike (S), envelope (E), membrane (M), nucleocapsid (N) proteins, and ORF1a/b polyproteins. Glycoprotein S is critical for the immune response and is the major antigen that elicits immunity through vaccination or recovery from COVID-19 [14]. It facilitates the attachment and entry of SARS-CoV-2 into host cells by binding to the angiotensin-converting enzyme 2 (ACE-2) receptor [4]. The primary role of NAbs is to prevent infection by binding to specific antigens, such as the S protein of SARS-CoV-2. By targeting key regions required for the virus to infect host cells, such as the receptor binding domain (RBD), NAbs block the virus from entering cells. In this way, they can effectively neutralize its ability to cause disease [15]. NAbs against SARS-CoV-2 exhibit variable persistence in the bloodstream, influenced by factors such as vaccination, infection, age, and booster doses.

Various longitudinal studies have investigated the persistence of neutralizing antibodies in serum samples, their ability to maintain effectiveness over time, and their response to newly emerging variants. Some studies have specifically analyzed differences between vaccinated, previously infected, and hybrid immunity (vaccinated and infected) cohorts [10,16,17,18]. In this study, ‘hybrid immunity’ refers to immune responses resulting from a combination of mRNA vaccination and subsequent SARS-CoV-2 infection. Although vaccine-induced immunity offers strong initial protection, antibody levels decline over time. Booster doses have been shown to restore and broaden neutralizing responses, particularly in vulnerable populations with underlying conditions or immunodeficiency [19,20,21].

During the COVID-19 pandemic, pseudotyped viruses became widely used as safe and standardized alternatives to live SARS-CoV-2 for studying viral entry and evaluating vaccine efficacy via pseudotype-based neutralization assays. Their main advantage lies in eliminating the need for BSL-3 laboratories, as the assays can be safely conducted under BSL-2 conditions in a rapid and high-throughput manner. To monitor the neutralization ability of sera samples, we used neutralization assays based on the recombinant vesicular stomatitis virus (rVSV) pseudotyped system, which can imitate the SARS-CoV-2 virus by coating its surface with the S protein of the targeted variant. rVSV is replicable deficient (rVSV∆G) because it lacks the gene for the G protein, which is necessary for replication [22,23,24].

The long-term dynamics and persistence of the elicited neutralizing antibody responses are critical factors in understanding the durability of protection and informing future vaccination strategies against both the original virus and emerging variants. This study aims to provide a comprehensive picture of the dynamics of neutralizing antibodies generated after vaccination and recovery from COVID-19. The obtained data provide a valuable addition to the body of information on the long-term neutralizing capacity of antibodies to SARS-CoV-2.

## 2. Materials and Methods

### 2.1. Participants

**Figure 1:**
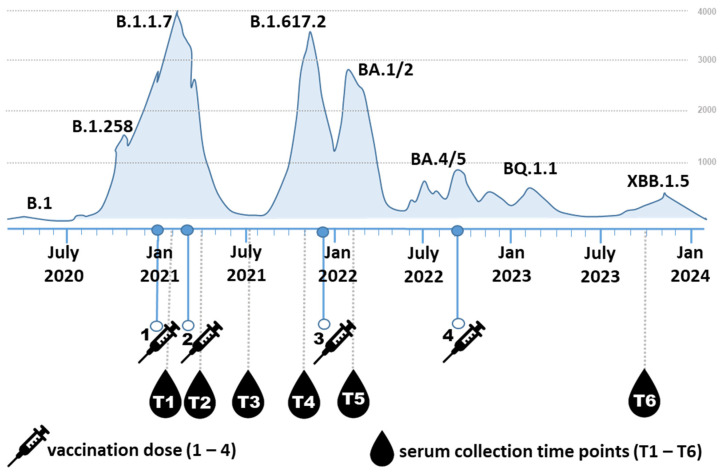
Schematic representation of the experimental timeline. The timeline illustrates the progression of dominant SARS-CoV-2 variants in Slovakia and their association with fluctuations in COVID-19 hospitalizations [25]. Major infection waves correspond to the emergence of key variants, including B.1.1.7, B.1.617.2, BA.1/2, BA.4/5, BQ.1.1, and XBB.1.5. Vaccination events are indicated by syringe icons (doses 1–4), and serum collection time points (T1–T6) are marked by drop icons. The six sampling time points correspond approximately to: T1—3 weeks after the first vaccine dose, T2—1-month post second vaccine dose, T3—3–4 months post second vaccine dose, T4—6–8 months post second vaccine dose, T5—2 weeks post third dose, T6—2 years post third dose (31 months post first vaccine dose).

Participants (5M/14F) aged 26–59 years (mean 41.4 ± 9.4 years; median 44.0 years) were voluntarily recruited from the employees of the Biomedical Research Center of the Slovak Academy of Sciences. Blood serum samples were collected from 19 individuals who had received at least three doses of the COVID-19 mRNA vaccine Comirnaty (Pfizer/BioNTech) between 2021 and 2023. Three participants also received a fourth dose during the study period. Each participant provided six serum samples, totaling 114 serum samples. Blood was drawn at six time points (Figure 1): (T1) three weeks after the first vaccine dose, (T2) approximately one month after the second dose, (T3) three to four months after the second dose, (T4) six to eight months after the second dose, (T5) two weeks after the third dose, and (T6) two years after the third dose.

Between these time points, 15 participants contracted COVID-19 once or twice, caused by different SARS-CoV-2 variants. Among the 15 participants who contracted SARS-CoV-2, 13 were infected between timepoints T5 and T6, and two between T4 and T5. Thirteen of these infections occurred between T5 and T6, and two between T4 and T5. Only four respondents (all with three vaccine doses) reported never having been infected. The dates of positive PCR tests, along with dates of vaccinations, identified variants, and course of disease, are listed in Supplementary Table S1. The severity of COVID-19 symptoms was categorized as very mild, mild, or moderate. Very mild cases included no fever or fever up to 38 °C, with symptoms lasting 3 to 5 days. Mild cases involved fever above 38 °C or prolonged low-grade fever, possibly accompanied by additional symptoms such as fatigue or joint pain, and lasted approximately 7 days. Moderate cases were characterized by high fever and more severe symptoms persisting for more than 7 days. The course of the pandemic in Slovakia, along with the indicated vaccination and blood collection time points, is summarized in Figure 1.

### 2.2. Plasmids

Plasmids carrying sequences of the S protein from various SARS-CoV-2 variants were generously provided by Addgene and David Nemazee (RRIDs: Addgene_170442, 170451, 172320, 180375, 194494, 194493), Alejandro Balazs (169467), and Marceline Côté (18603).

### 2.3. Cell Lines

We used BHK21 cells (ATCC, CCL-10), Vero E6 cells stably expressing human ACE2 and TMPRSS2 (BEI NR-54970) and 293FT cells stably expressing T7 polymerase and VSVG (293FT-VSVG-T7pol) [26] using the Sleeping Beauty transposon plasmid expression system [27] kindly provided by Dr. Ivan Kosik, Dr. Thiasheng Li, and Dr. Jon Yewdell (NIAID, NIH, USA). All cell lines were maintained in low-glucose Dulbecco’s Modified Eagle Medium (Biosera, Cholet, France) supplemented with 10% Fetal Bovine Serum (FBS) (Gibco), 100 µg/mL Penicillin–Streptomycin–Amphotermicin B (PSA), 50 µg/mL Gentamicin Sulfate (Thermo Fisher Scientific Inc., Waltham, MA, USA).

### 2.4. SARS-CoV-2 Spike Protein Pseudotype-Based Neutralization Assay

Recombinant rVSVΔG-GFP-G virus (Kerafast) was prepared in 293T7-VSVG cells (MOI = 5). Viral titration was performed on BHK21 cells as described previously [23,24]. To produce SARS-CoV-2 pseudotyped viruses, BHK21 cells were transfected with S protein-encoding plasmids using TurboFect (Thermo Fisher Scientific) and incubated for 24 h at 37 °C. Cells were then infected with rVSVΔG-GFP-G (MOI = 5) and incubated overnight. Anti-VSV-G antibody (1 µg/mL) was added to neutralize residual parental virus. rVSVΔG-GFP-S particles were harvested upon GFP expression and cytopathic effect after 24–48 h, and stored at –80 °C [23,24,28].

For the pseudotype-based neutralization assay, Vero E6 ACE2/TMPRSS2 cells (19,000/well) were seeded in 96-well plates. Sera were diluted two-fold in serum-free DMEM, mixed with pseudovirus (MOI = 0.1), and incubated for 1 h at 37 °C. The mixtures were transferred to cell monolayers and incubated for 20–24 h. Neutralization was assessed by GFP fluorescence microscopy; ImageJ (version 1.54d) was used to quantify fluorescent foci. Infection rates were normalized to positive controls, and NT_50_ values were calculated using the log-logistic model in R software (version 4.4.3) in ‘drc’ package (version 3.0-1) [29]. Plots were generated with ‘ggplot2’ [30]. A detailed description of the whole procedure is provided in the Supplementary methods.

### 2.5. Anti-SARS-CoV-2 S1 IgG Enzyme-Linked Immunosorbent Assay (ELISA)

To assess antibody responses, we used a semiquantitative anti-SARS-CoV-2 IgG ELISA targeting the S1 subunit of the SARS-CoV-2 spike protein (EuroImmun Medizinische Labordiagnostika AG, Lübeck, Germany) according to the manufacturer’s instructions. Results were expressed as sample/calibrator OD ratios. According to manufacturer criteria, ratios < 0.8 were negative, 0.8–1.0 borderline, and >1.1 positive. Borderline values were considered positive, as in Kajanová et al. [31,32].

### 2.6. Statistical Analysis

The 50% neutralization titer (NT_50_) was determined using a four-parameter log-logistic dose–response model implemented in the drc package in R software. The dose–response model (drm) function was used to fit the model, and the ED50() extracted NT_50_ values. Neutralization curves were visualized using ggplot2, with fitted curves generated by geom_smooth.

Statistical analyses of the obtained data were performed using GraphPad Prism 10.4.1. (Boston, MA, USA) and IBM SPSS Statistics version 26 (SPSS Inc., Chicago, IL, USA). The data were tested for normal distribution using the Shapiro–Wilk test and the appropriate statistical tests were then used. Changes in antibody levels over time were analyzed using the repeated measures ANOVA with a Bonferroni post hoc test. The paired *t*-test was used to analyze the change in antibody levels between T5 and T6 in infected and uninfected subgroups of volunteers. Pearson or Spearman correlation coefficients r with 95% CI were calculated to examine the association of anti-S1 IgG antibody levels and neutralizing antibody titers. All values are expressed as mean ± standard deviation unless stated otherwise. A *p*-value of less than 0.05 was considered statistically significant.

## 3. Results

### 3.1. Correlation of Anti-SARS-CoV-2-S1 IgG Antibodies with Neutralization Titers

**Figure 2:**
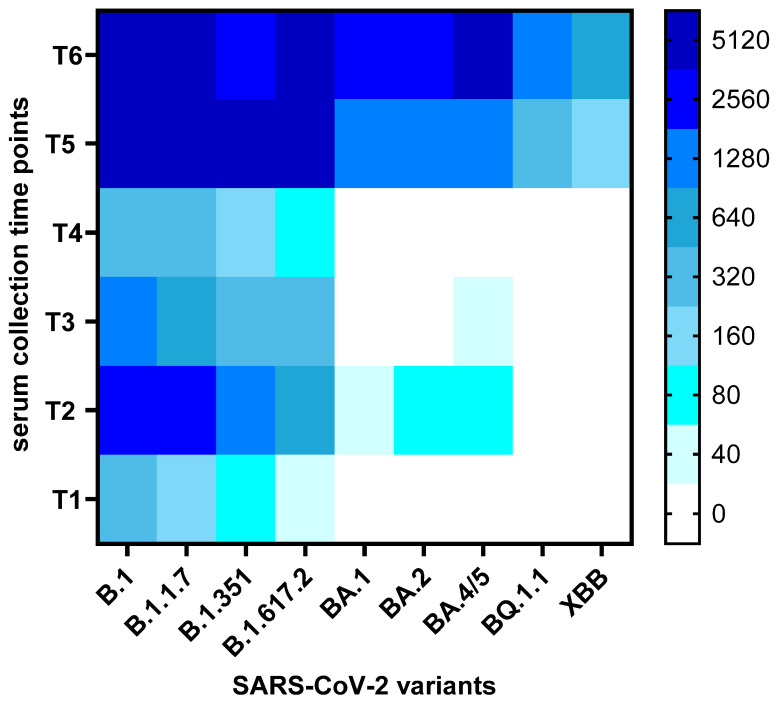
Heatmap of neutralization titers across SARS-CoV-2 variants over time. The heatmap displays average neutralization titers (NT_50_) against nine SARS-CoV-2 variants across six serum collection time points (T1–T6). Each square represents the mean NT_50_ value for a given variant and time point. Dark blue indicates high neutralization capacity, lighter shades reflect lower titers, and white denotes the absence of detectable neutralization.

Altogether, a total of 114 serum samples from 19 participants were analyzed for the presence of neutralizing antibodies to nine SARS-CoV-2 variants and sub-variants (B.1, B.1.1.7, B.1.617.2, B.1.351, BA.1, BA.2, BA.4/5, BQ.1.1, XBB). Thus, this study assessed the presence of neutralizing antibodies against virus variants that had not yet emerged at the time of serum collection (e.g., BA.1, BA.2, BA.4/5, BQ.1.1, XBB for the first five sample collections). The overall changes in neutralization titers against the nine variants over time are presented as a heatmap of mean titers in Figure 2, while the individual kinetics of neutralization titers for each participant are shown in Supplementary Figure S1.

**Figure 3:**
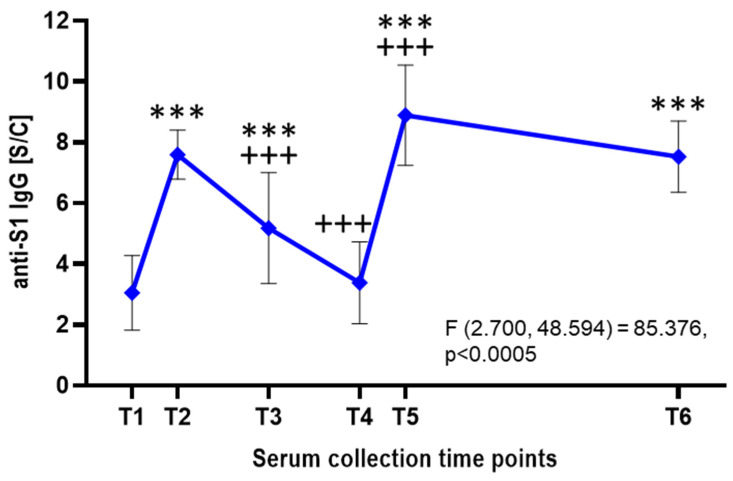
Detection of SARS-CoV-2 anti-S1 IgG by ELISA. The graph displays anti-S1 IgG antibody levels expressed as sample-to-calibrator (S/C) ratios, measured by ELISA at six time points. Blue points indicate mean values with standard deviations as error bars (n = 19). A repeated-measures ANOVA with Greenhouse–Geisser correction showed a statistically significant difference in anti-S1 IgG levels across time points (F(2.700, 48.594) = 85.376, *p* < 0.0005). Post hoc analysis with Bonferroni adjustment revealed significantly higher IgG levels at T2, T3, T5, and T6 compared to T1 (*p* < 0.001). No significant difference was observed between T5 and T6 (*p* = 0.215), nor at T4 compared to T1. *** *p* < 0.001 for time point vs. T1; +++ *p* < 0.001 for time point vs. the preceding sampling time point.

Besides neutralization assays, all serum samples were analyzed by ELISA-detecting IgG antibodies targeting the S1 subunit of the SARS-CoV-2 S protein (Figure 3). Following the first vaccine dose, the mean anti-S1 IgG antibody level was approximately 3.05 ± 1.23 S/C, indicating a relatively low response. After the second dose, antibody levels rose significantly (*p* < 0.001) to an average of 7.6 ± 0.81 S/C within a few weeks. However, a gradual decline followed, with levels dropping to an average of 5.18 S/C after 3–4 months and 3.38 ± 1.35 S/C after 6–8 months. A significant increase (*p* < 0.001) was observed after the third vaccine dose, with antibody levels peaking at 8.89 ± 1.65 S/C. These levels remained relatively stable over the next two years, averaging 7.53 ± 1.17 S/C (Figure 3).

**Figure 4:**
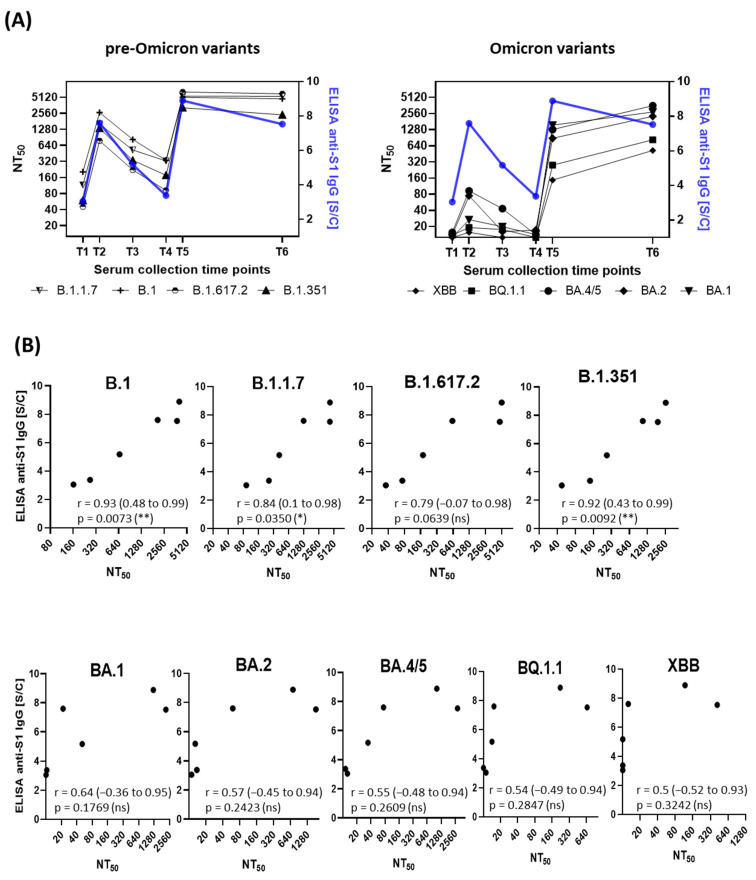
Correlation between anti-S1 IgG antibody levels and neutralization titers. (**A**) Dual *y*-axis plots illustrate the temporal relationship between anti-S1 IgG antibody levels (blue line, right *y*-axis) and neutralization titers (NT_50_, black lines, left *y*-axis) across six time points. The left *y*-axis shows the arithmetic mean of neutralization titers for each SARS-CoV-2 variant, while the right *y*-axis (blue line) represents the mean anti-S1 IgG levels. (**B**) Correlation plots between NT_50_ values and anti-S1 IgG levels for each variant. Pearson correlation coefficients (r) with 95% confidence intervals and *p* values are shown. Asterisks show statistical significance: *p* < 0.05 (*); *p* < 0.01 (**); *p* > 0.05 (ns, not significant).

Pearson correlation analysis was conducted to evaluate the relationship between mean neutralizing antibody titers and anti-S1 IgG antibody levels measured by ELISA (Figure 4). The calculated correlation coefficients (r) showed significant differences between SARS-CoV-2 variants. For pre-Omicron variants, a strong correlation was observed (B.1, r = 0.93; B.1.1.7, r = 0.84; B.1.617.2, r = 0.79; B.1.351, r = 0.92), reflecting a consistent relationship between antibody titers and neutralization potential. In contrast, for Omicron variants, correlation coefficient values around 0.5 indicated a moderate correlation (BA.1, r = 0.64; BA.2, r = 0.57; BA.4/5, r = 0.55; BQ.1.1, r = 0.54; XBB, r = 0.50). These findings suggest that the extensive accumulation of novel mutations in Omicron variants significantly impacts viral neutralization, particularly when immunity is induced through vaccination against the genuine B.1 variant.

### 3.2. Neutralization Trends Across Virus Variants

**Figure 5:**
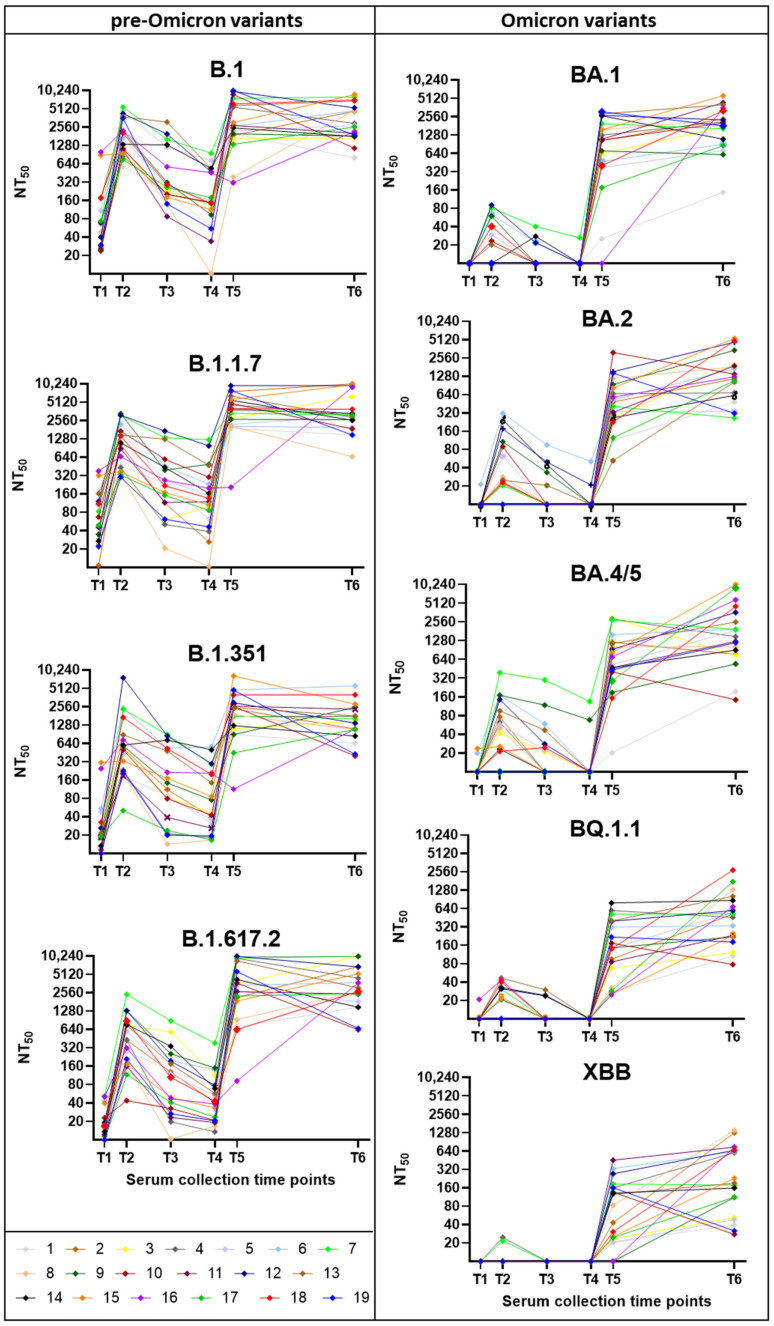
Neutralization assays for nine SARS-CoV-2 variants. Each graph shows results from pseudotype-based neutralization assays performed for a single SARS-CoV-2 variant, using 114 serum samples collected at six time points from 19 participants (n = 19 per time point). The left column presents results for pre-Omicron variants (B.1, B.1.1.7, B.1.351, B.1.617.2), while the right column displays Omicron subvariants (BA.1, BA.2, BA.4/5, BQ.1.1, XBB). Neutralization titers are expressed as NT_50_ values.

The neutralization capacity varied markedly depending on the vaccination dose and prior SARS-CoV-2 infections and analyzed variants (Figure 5). The weakest neutralization was observed after the first vaccine dose (T1). A substantial (*p* < 0.01) increase in neutralization capacity followed the second dose, with serum samples from the T2 effectively neutralizing pre-Omicron variants (B.1, B.1.1.7, B.1.617.2) with a non-significant increase in neutralization capacity for B.1.351 and the Omicron variants (BA.1, BA.2, BA.4/5, BQ.1.1; XBB). Three to four months after the second dose (T3), neutralization capacity significantly declined for pre-Omicron variants, while showing limited ability to neutralize Omicron variants. Six to eight months post-second dose (T4), neutralization capacity further decreased for pre-Omicron variants, with no detectable activity against Omicron variants. Two to four weeks after the third dose (T5), neutralization titers markedly increased (*p* < 0.01–*p* < 0.001), particularly against pre-Omicron variants, but also showed a noticeable improvement for all Omicron variants (*p* < 0.01). By two years post-third dose (T6), neutralization titers had relatively stabilized and did not show such a significant decline as three months after the second vaccine dose. The serum samples from the last collection (2 years post-3rd vaccine dose, T6) showed the overall highest neutralization capacity across all variants. At that time, most of the respondents experienced at least one clinically noticeable COVID-19 attributable to Omicron infections (n = 13) or Delta and then Omicron infections (n = 2), while only four reported no infection. The lowest neutralization capacity was observed against the XBB variant, a recombinant virus of two BA.2 descendants, BJ.1 and BM.1.1.1 [33].

**Figure 6:**
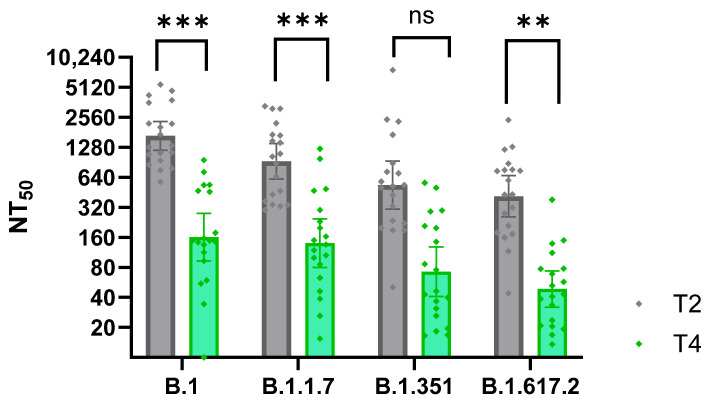
Decline in neutralization capacity of sera following the second vaccine dose. The graph compares neutralization titers (NT_50_) of serum samples collected shortly after the second vaccine dose (T2) and 6–8 months later (T4) from 19 participants (n = 19). Pseudotype-based neutralization assays were performed against four SARS-CoV-2 variants. Repeated measures ANOVA with Bonferroni post hoc test revealed statistically significant declines in B.1, B.1.1.7, *p* < 0.001 (***); B.1.617.2, *p* < 0.01 (**); ns, not significant, *p* > 0.05.

The most substantial drop in the neutralization ability was observed in the sera taken 6–8 months (T4) after the second vaccine dose in comparison with the sera taken 1 month after the second vaccine dose (T2; Figure 6). This decline, occurring just before the arrival of the Delta wave, is also visible in the overall reduction in anti-S1 IgG antibody levels (Figure 4A). In contrast, after the third vaccine, even after two years (T6), and even for those without clinical disease, neutralization titers did not show such a marked decline as three months after the second vaccine dose. These findings confirm the importance of the 3rd (booster) vaccine dose in sustaining long-lasting neutralizing immunity.

**Figure 7:**
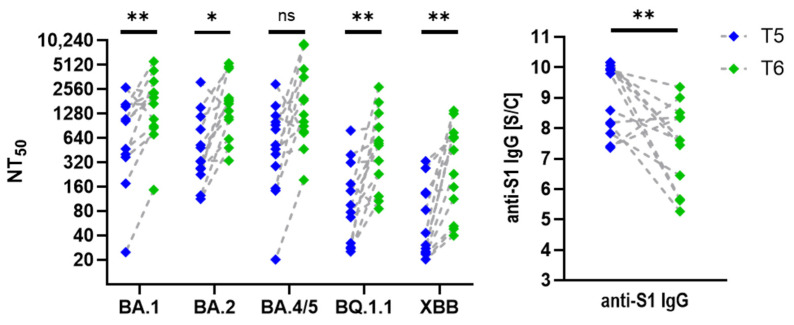
Neutralization of Omicron variants using serum samples collected at T5 and T6. Graphs show changes in neutralization titers (NT_50_) against Omicron variants and corresponding anti-S1 IgG antibody levels between T5 (2 weeks post-third dose) and T6 (31 months post-first dose) in participants who experienced Omicron infection during the study period (n = 13). Pairwise comparison revealed statistically significant increases in BA.1, BQ.1.1, XBB, *p* < 0.01 (**); BA.2, *p* < 0.05 (*), and a decline in anti-S1 IgG, *p* < 0.01 (**). *p* > 0.05 (ns, not significant).

Interestingly, participants vaccinated three times and infected at least once with an Omicron variant displayed slightly higher neutralization titers in their most recent serum collection (T6), despite a significant decline (*p* = 0.002) in anti-S1 IgG levels (Figure 7). In individuals who received three vaccine doses but did not contract SARS-CoV-2, neutralization titers against Omicron variants did not increase as markedly as in those who experienced clinical infection but nevertheless remained stable over the two-year period following the third dose. Trends highlight the effect of hybrid immunity versus vaccination alone on long-term neutralization capacity and antibody levels.

To further explore whether the elevated antibody levels observed at T6 reflect the lingering effects of recent infections or represent genuine long-term persistence, we examined the temporal relationship between the last COVID-19-related event and anti-S1 IgG levels. Specifically, we analyzed the number of weeks elapsed (range: 3–103 weeks, mean ± SD: 68.4 ± 26.6 weeks) since either the most recent PCR-confirmed SARS-CoV-2 infection or the last vaccine dose—whichever occurred closer to the T6 time point—and correlated this with anti-S1 IgG levels at T6. No significant time-dependent association was found (*p* = 0.121), and there was no difference in antibody levels between individuals with an event within the previous 52 weeks (n = 6; 8.03 ± 1.46 S/C) and those with more distant events (7.29 ± 1.00 S/C; *p* = 0.210). These findings suggest that the elevated antibody levels at T6 are not primarily a consequence of recent infections but rather reflect sustained humoral responses maintained over the long term.

In summary, a significant increase in neutralization titers was observed after the second and third doses, particularly against pre-Omicron variants. In contrast, titers against Omicron subvariants remain much lower or even undetectable, highlighting strong immune evasion. Nevertheless, the third vaccine dose and natural infection contributed to improved long-term immunity, as evidenced by sustained titers at the two-year mark.

## 4. Discussion

This study provides a comprehensive longitudinal analysis of the humoral immune response following mRNA vaccination and natural SARS-CoV-2 infection. By assessing neutralizing antibody titers against multiple SARS-CoV-2 variants, including those that had not yet emerged at the time of serum collection, we gained valuable insights into the durability and breadth of vaccine-induced immunity.

Our findings highlight the robust induction of anti-S1 IgG antibodies and neutralization capacity following the second and third vaccine doses. A significant rise in neutralizing titers was observed after the second dose, with effective neutralization of pre-Omicron variants. However, these titers declined substantially over time, particularly three to six months post-vaccination. This waning immunity coincided with the emergence of the Delta variant, suggesting that declining neutralization titers may have contributed to increased susceptibility to infection during this period. These results are consistent with other studies demonstrating the transient nature of vaccine-induced antibody responses, emphasizing the need for booster doses to sustain immunity [34,35,36].

The third vaccine dose significantly boosted neutralization titers, not only against pre-Omicron variants but also against Omicron subvariants. Notably, despite an initial increase in anti-S1 IgG levels post-booster, these levels gradually declined over two years. However, neutralization titers remained relatively stable, in the case of Omicron variants, even increased, suggesting a qualitative improvement in antibody response, possibly due to affinity maturation and long-lived plasma cell contributions [37,38]. The sustained neutralization titers even two years post-booster reinforce the importance of the third vaccine dose in maintaining durable immune protection.

A key observation was the differential response among participants based on infection history. Those who had received three vaccine doses and experienced an Omicron infection displayed slightly higher neutralization titers at the final time point (T6) compared to those who were triple-vaccinated but infection-naïve. This confirms that hybrid immunity may provide superior and more sustained protection against emerging variants. Similar findings have been reported in multiple independent studies, which demonstrated that repeated antigen exposure through infection following vaccination enhances both the breadth and durability of the immune response. For example, Andreano et al. [39] showed that hybrid immunity improves B cell maturation and antibody quality, while Goel et al. [40] observed distinct antibody and memory B cell profiles in hybrid-immune individuals. A meta-analysis by Bobrovitz et al. [41] further confirmed that hybrid immunity provides stronger protection against Omicron infection and severe outcomes than vaccination alone.

Our correlation analysis revealed a strong association between anti-S1 IgG levels and neutralization titers for pre-Omicron variants (r = 0.79–0.93; *p* < 0.05), but a more moderate correlation for Omicron subvariants (r ≈ 0.50–0.64; ns). This reduced correlation suggests that while IgG levels remain a useful marker of immunity, they may not fully predict neutralization efficacy against highly mutated variants. The substantial immune evasion observed in Omicron variants underscores the necessity of updated vaccines in fully preventing infection with these later-emerging lineages [42,43,44].

We observed the lowest neutralization capacity against the XBB variant [33]. This observation is consistent with previous findings highlighting its significant immune evasion properties. For instance, Wang et al. [45] reported that neutralizing antibody titers against XBB subvariants were reduced by 66- to 155-fold compared to earlier strains, indicating a substantial decrease in vaccine-induced antibody effectiveness. Additionally, Zhang et al. [46] demonstrated that individuals previously infected with earlier Omicron subvariants exhibited markedly reduced neutralizing antibody levels against several XBB subvariants, suggesting limited cross-protection provided by prototype- and Omicron BA.5 variant-induced neutralizing antibodies.

Interestingly, our findings align with prior studies demonstrating that vaccine-induced sera collected before the emergence of Omicron variants exhibited measurable cross-neutralizing activity against BA.1 and BA.2 sublineages [47,48]. Andreano et al. [47] identified both receptor binding domain (RBD)- and M-terminal domain (NTD)-targeting monoclonal antibodies capable of neutralizing Omicron variants, suggesting that conserved epitopes—particularly in Class 3 regions and specific NTD sites—can elicit broadly reactive antibodies even in the absence of prior exposure to those variants. Gruell et al. [48] further showed that although primary vaccination induced only weak serum neutralization of Omicron, a third mRNA vaccine dose dramatically enhanced neutralizing titers, supporting the concept of affinity maturation leading to broader cross-reactivity. Consistent with these reports, we observed higher neutralization titers against BA.2 than BA.1, a pattern previously linked to epitope accessibility and structural differences between subvariants.

Despite these valuable insights, our study has some limitations. The relatively small sample size (n = 19) and the homogenous nature of the cohort—composed of participants from a single research institution—may limit the generalizability of our findings to broader populations. This limitation was also evident when interpreting subgroup differences, as the number of participants without documented infection was too small for robust statistical analysis. Furthermore, while pseudotyped virus assays are widely used and enable safe, high-throughput assessment of neutralizing activity under BSL-2 conditions, they may not fully replicate the complexity of live virus infection. Multiple studies have demonstrated a high level of correlation between pseudotyped and authentic virus neutralization assays, supporting their utility as surrogates for live virus assays [49,50,51,52]. However, discrepancies in sensitivity and neutralization potency have been reported, particularly when evaluating highly immune-evasive variants such as Omicron subvariants [53,54].

Furthermore, while we focused on humoral responses, cellular immunity was not assessed. T cell-mediated responses play a crucial role in long-term protection, particularly against immune-evasive variants. Multiple studies have shown that T cell responses remain robust across variants and persist long after antibody waning [4,5], likely contributing to protection even when neutralizing antibody levels decline. Complementary data on T cell immunity would help provide a more complete understanding of vaccine-induced protection.

However, a notable strength of our study is its extensive follow-up period of two years, which provides a detailed perspective on the long-term dynamics of the immune response. Many longitudinal studies on SARS-CoV-2 vaccine-induced immunity have been limited to shorter durations, often ranging from six months to one year. Several other long-term studies, such as the ProHEpiC-19 study (24 months, 800 healthcare workers) [55], the PARIS study (3 years, large cohort of healthcare workers) [56], or a 20-month study on recovered individuals of Hvidt et al. [57], have also provided valuable insights into SARS-CoV-2 long-term immunity. However, these studies primarily focused on infection risk, antibody presence, and broad immune responses rather than detailed neutralization analyses across multiple variants, as conducted in our study incorporating extensive variant-specific neutralization testing. Our extended follow-up allows for a more comprehensive assessment of the durability of vaccine-induced and hybrid immunity over an extended period, offering valuable insights into the long-term effectiveness of vaccination strategies.

## 5. Conclusions

In conclusion, our study reinforces the importance of booster vaccinations in maintaining neutralization capacity and highlights the added benefit of hybrid immunity. While vaccine-induced protection wanes over time, natural infection following vaccination appears to enhance long-term immunity. These findings support ongoing efforts to optimize booster strategies and adapt vaccines to emerging variants to ensure continued protection against SARS-CoV-2.

## Data Availability

The original contributions presented in this study are included in the article/Supplementary Materials. Further inquiries can be directed to the corresponding author.

## Supplementary Materials

The following supporting information can be downloaded at https://www.mdpi.com/article/10.3390/v17050675/s1, Supplementary Table S1: Basic Characteristics of Study Participants. Demographic information, vaccination history, and SARS-CoV-2 infection details for each participant, including variant type and clinical course of the disease. Supplementary Table S2: SARS-CoV-2 Anti-S1 IgG Antibody Levels. ELISA-based measurements of anti-S1 IgG antibody levels (expressed as sample-to-calibrator [S/C] ratios) in serum samples collected from participants at six defined time points during the study. Supplementary Figure S1: Pseudotype-Based Neutralization Assays. Each panel displays neutralization assay results for individual participants, showing serum-neutralizing activity across six time points against nine distinct SARS-CoV-2 variants using a pseudotype-based system. Supplementary methods: SARS-CoV-2 Spike protein Pseudotype-based neutralization assay.
