# Supplementary material for: Long-Term Dynamics of SARS-CoV-2 Variant-Specific Neutralizing Antibodies Following mRNA Vaccination and Infection
Source: Viruses. 2025 May 6;17(5):675. doi: 10.3390/v17050675 (PMC12115524; doi:10.3390/v17050675)
Supplement: Supplementary file 1 [file viruses-17-00675-s001.zip › viruses-3607226-supplementary.pdf]

Supplementary Table S1: Basic Characteristics of Study Participants. Demographic information, vaccination history, and SARS-CoV-2 infection details for each participant, including variant type and clinical course of the disease.

participant	age	gender	1. dose	2. dose	3. dose	4. dose	COVID-19	variant	course of the disease
1	44	male	12/1/21	9/2/21	28/10/21	25/10/22	29/3/22	Omicron	mild
2	57	male	12/1/21	9/2/21	4/11/21	no	12/01/23	Omicron	very mild
3	59	female	12/1/21	9/2/21	4/11/21	no	3/3/22	Omicron	moderate
4	36	female	9/2/21	8/3/21	11/2/22	30/10/22	26/10/21	Delta	moderate
5	32	female	8/2/21	8/3/21	12/1/22	no	17/3/22	Omicron	very mild
6	27	female	14/1/21	11/2/21	3/12/21	no	28/9/22	Omicron	mild
7	26	female	22/1/21	19/2/21	3/12/21	no	no		-
8	35	female	14/1/21	11/2/21	21/11/21	no	8/22	Omicron	mild
9	30	female	14/1/21	11/2/21	26/11/21	no	no		-
10	45	female	22/1/21	19/2/21	28/1/2022	no	31/1/22; 3/3/22	Delta + Omicron	moderate/ very mild
11	45	female	20/1/2021	17/2/2021	20/11/21	no	2/22<br>9/2022	Delta + Omicron	mild/ very mild
12	50	female	14/1/2021	11/2/2021	26/1/2022	no	10/2022	Omicron	very mild
13	32	male	9/2/2021	8/3/2021	no	no	17/1/2022	Delta	mild
14	45	female	12/1/2021	9/2/2021	26/11/2021	no	3/2022	Omicron	mild
15	41	male	17/2/2021	17/3/2021	9/12/2021	no	29/3/2022; 28/9/2023	Omicron + Omicron	mild/ very mild
16	48	female	8/1/2021	29/1/2021	13/11/2021	no	30/10/2022	Omicron	mild
17	46	male	8/1/2021	29/1/2021	28/10/2021	no	no		-
18	40	female	4/1/21	25/1/21	28/10/21	25/10/22	4/2022; 12/2023	Omicron + Omicron	mild
19	48	female	8/1/21	29/1/21	20/11/21	no	no		-

Supplementary Table S2: SARS-CoV-2 Anti-S1 IgG Antibody Levels. ELISA-based measurements of anti-S1 IgG antibody levels (expressed as sample-to-calibrator [S/C] ratios) in serum samples collected from participants at six defined time points during the study.

participant	Anti-S1 IgG [S/C]					
	**T1**	**T2**	**T3**	**T4**	**T5**	**T6**
1	2.68	6.38	3.88	2.63	8.14	5.27
2	1.75	7.53	4.28	3.2	9.91	9.36
3	1.75	7.44	5.9	2.80	9.96	7.45
4	1.76	7.59	2.48	1.34	9.97	7.84
5	3.29	7.83	6.1	5.00	8.59	7.61
6	4.6	7.57	7.88	5.35	10.16	7.63
7	5.44	8.89	7.21	6.4	8.33	8.65
8	1.31	5.65	1.61	1.2	7.84	6.45
9	3.59	7.29	6.30	3.51	9.55	7.43
10	2.7	7.61	4.69	2.41	10.17	5.66
11	3.28	7.71	4.87	2.61	10.6	7.63
12	5.33	8.73	8.51	5.22	9.92	8.52
13	2.85	7.68	7.13	4.42	9.81	8.1
14	1.48	7.78	6.10	3.53	9.8	5.63
15	4.5	7.78	4.31	3.00	7.36	9.1
16	2.92	7.68	5.55	3.33	8.19	8.37
17	2.58	6.77	5.12	3.68	3.49	7.93
18	2.61	7.26	4.71	3.44	7.41	8.35
19	1.5	9.2	2.64	1.88	10.27	6.21

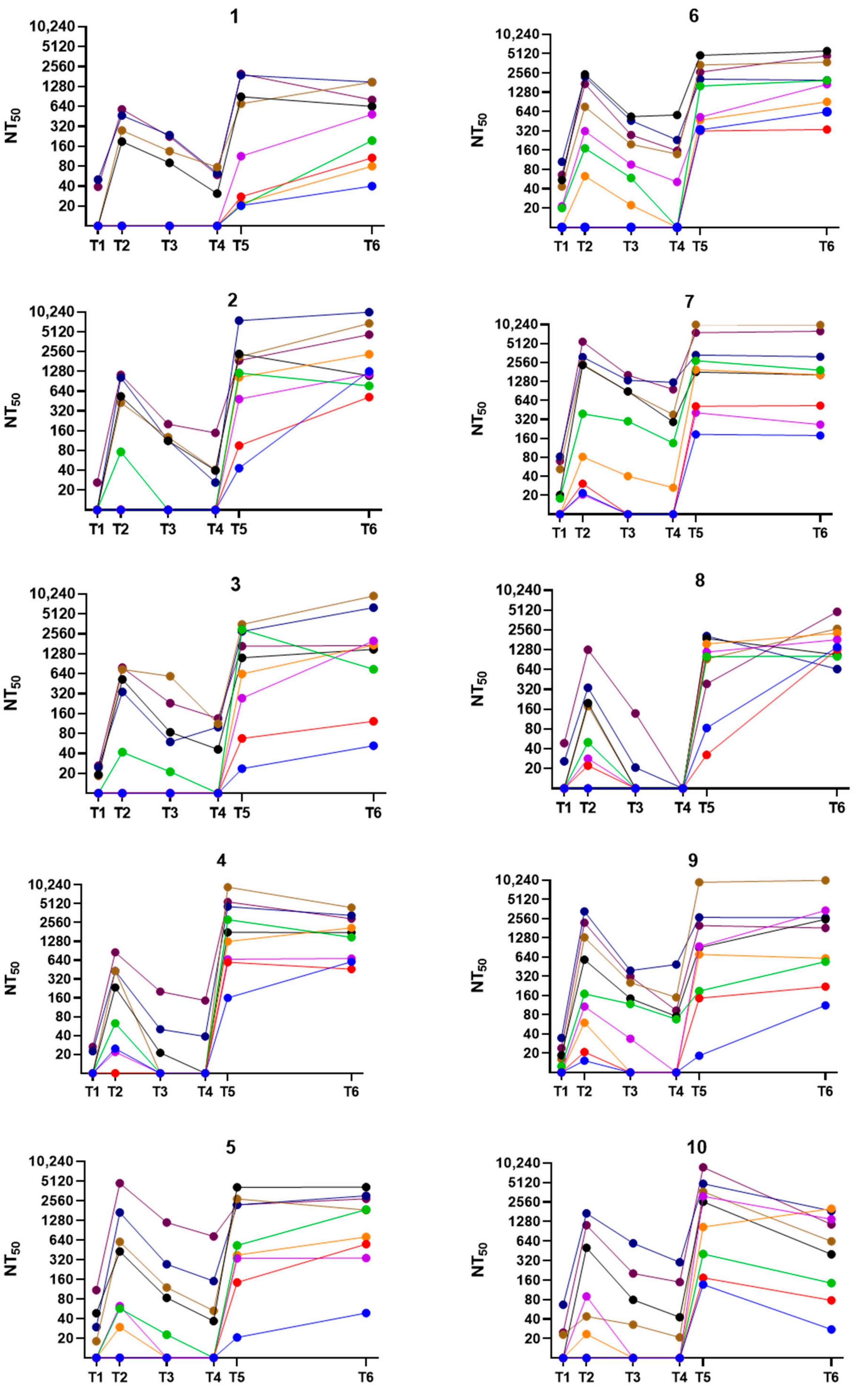
1
6
2
7
3
8
4
9
5
10
NT50
10,240
5120
2560
1280
640
320
160
80
40
20
T1
T2
T3
T4
T5
T6

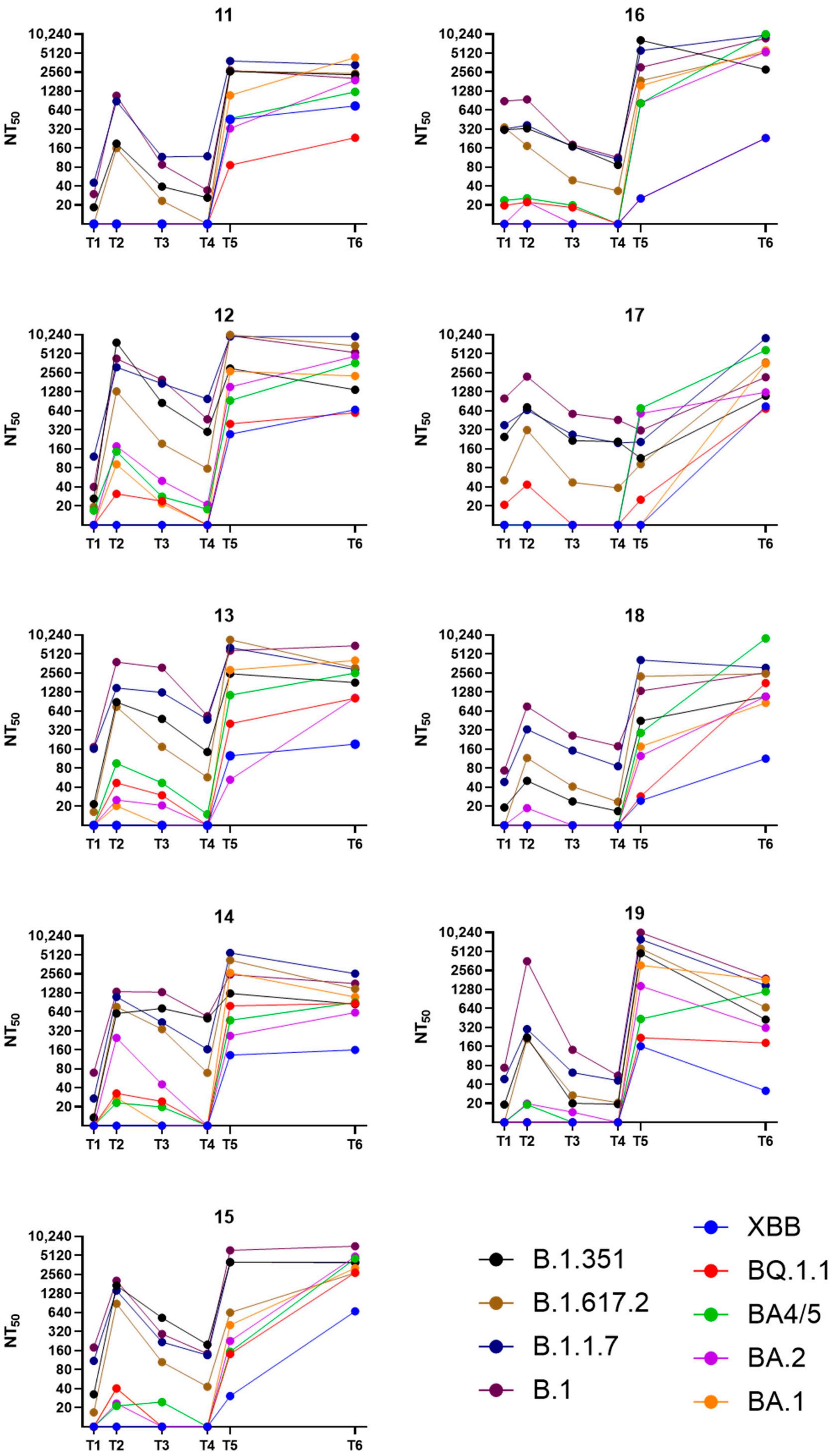
11
12
13
14
15
16
17
18
19
NT50
10,240
5120
2560
1280
640
320
160
80
40
20
T1
T2
T3
T4
T5
T6
B.1.351
B.1.617.2
B.1.1.7
B.1
XBB
BQ.1.1
BA4/5
BA.2
BA.1

Supplementary Figure S1: Pseudotype-Based Neutralization Assays. Each panel displays neutralization assay results for individual participants, showing serum neutralizing activity across six time points against nine distinct SARS-CoV-2 variants using a pseudotype-based system.

Supplementary methods

SARS-CoV-2 Spike protein Pseudotype-based neutralization assay

Generation of rVSVΔG-GFP-G stock

To construct an rVSV-based pseudotyped virus, a recombinant vesicular stomatitis virus (rVSV) lacking the gene encoding the G protein (rVSVΔG) and carrying a GFP reporter gene was used (Kerafast, Newark, CA, USA). For continuous production, rVSVΔG-GFP-G particles were amplified by infecting 293T7 VSVG cells at a multiplicity of infection (MOI) of 5. The resulting rVSVΔG-GFP-G stock was stored at -80°C. Viral titration was performed on BHK21 cells to quantify the virus, as described previously (Nie et al., 2020; Condor Capcha et al., 2021).

Cell transfection and production of rVSVΔG-GFP-S

SARS-CoV-2 pseudotyped viruses were prepared by SARS-CoV-2 S protein expression plasmid transfection, providing membrane proteins on the cell surface, and rVSVΔG GFP G infection providing genomes of VSV. BHK21 cells were transfected with the plasmids carrying a sequence of the S protein with various variants (B:1, B.1.1.7, B.1.617.2, B.1.351, BA.1, BA.2, BA.4/5, BQ.1.1, XBB). Transfection was carried out with TurboFect Transfection Reagent (Thermo Fisher Scientific) according to the manufacturer's instructions, followed by 24 h incubation at 37°C and 5% CO_2. The next day, the infection with rVSVΔG-GFP-G was done at MOI = 5, and overnight incubation at 37°C, 5% CO_2 followed. Anti-VSV-G (Clone 8G5F11) (final concentration 1μg/ml) was added to the media to neutralise residual rVSVΔG-GFP-G. After 24 – 48 h, cells started showing VSV-induced cytopathic effects (CPE), and the cells expressed GFP. rVSVΔG-GFP-S stock was harvested and stored at -80°C (Nie et al., 2020; Xiong et al., 2020; Condor Capcha et al., 2021).

Pseudotype-based neutralisation assay

For pseudotype-based neutralisation assay, 19,000 Vero E6 ACE2 TMPRSS2 cells were seeded per well on a 96-well plate. Sera samples were diluted by using two-fold serial dilution in serum-free DMEM. A constant amount of viral particles (MOI = 0.1 per well) is introduced into the each diluted sera and incubated in thermostat at 37°C, and 5% CO2. for one hour. Subsequently, the serum-virus mixture was transferred to pre-prepared cell cultures, following the removal of residual serum, and further incubated for 20 – 24 hours in thermostat. Each neutralisation was done in duplicate for each dilution. The assay outcomes were assessed via fluorescence microscopy, with two representative images captured per well. Quantification of fluorescent foci was performed using ImageJ software, and the

percentage of infection was determined by normalising the number of GFP-expressing foci against the positive control. The 50% neutralisation titer (NT_{50}) was then calculated using the log-logistic dose-response model implemented in drc package within R software (Ritz et al., 2015). Plots were created using the ggplot2 package (Wickham, 2011).

References

Nie, J.; Li, Q.; Wu, J.; Zhao, C.; Hao, H.; Liu, H.; Zhang, L.; Nie, L.; Qin, H.; Wang, M.; et al. Quantification of SARS-CoV-2 Neutralizing Antibody by a Pseudotyped Virus-Based Assay. Nat. Protoc. 2020, 15, 3699–3715, doi:10.1038/s41596-020-0394-5.

Condor Capcha, J.M.; Lambert, G.; Dykxhoorn, D.M.; Salerno, A.G.; Hare, J.M.; Whitt, M.A.; Pahwa, S.; Jayaweera, D.T.; Shehadeh, L.A. Generation of SARS-CoV-2 Spike Pseudotyped Virus for Viral Entry and Neutralization Assays: A 1-Week Protocol. Front. Cardiovasc. Med. 2021, 7, 618651, doi:10.3389/fcvm.2020.618651.

Xiong, H.-L.; Wu, Y.-T.; Cao, J.-L.; Yang, R.; Liu, Y.-X.; Ma, J.; Qiao, X.-Y.; Yao, X.-Y.; Zhang, B.-H.; Zhang, Y.-L.; et al. Robust Neutralization Assay Based on SARS-CoV-2 S-Protein-Bearing Vesicular Stomatitis Virus (VSV) Pseudovirus and ACE2-Overexpressing BHK21 Cells. Emerg. Microbes Infect. 2020, 9, 2105–2113, doi:10.1080/22221751.2020.1815589.

Ritz, C.; Baty, F.; Streibig, J.C.; Gerhard, D. Dose-Response Analysis Using R. PLOS ONE 2015, 10, e0146021, doi:10.1371/journal.pone.0146021.

Wickham, H. Ggplot2. WIREs Comput. Stat. 2011, 3, 180–185, doi:10.1002/wics.147.
